# Engineered Hybrid Vesicles and Cellular Internalization in Mammary Cancer Cells

**DOI:** 10.3390/pharmaceutics16040440

**Published:** 2024-03-22

**Authors:** So Yun Kim, Dagyeong Guk, Youngdo Jeong, Eunji Kim, Hansol Kim, Sung Tae Kim

**Affiliations:** 1 Department of Nanoscience and Engineering, Inje University, Gimhae 50834, Republic of Korea; soyun1598@gmail.com (S.Y.K.); jac06234@daum.net (E.K.); hsk@inje.ac.kr (H.K.); 2 Center for Advanced Biomolecular Recognition, KIST Biomedical Research Division, Korea Institute of Science and Technology, Seoul 02792, Republic of Korea; dagyeong@kist.re.kr (D.G.); zerodegree@kist.re.kr (Y.J.); 3 KU-KIST Graduate School of Converging Science and Technology, Korea University, Seoul 02841, Republic of Korea; 4 HY-KIST Department of Bioconvergence, Hanyang University, Seoul 04763, Republic of Korea; 5 Department of Pharmaceutical Engineering, Inje University, Gimhae 50834, Republic of Korea

**Keywords:** extracellular vesicle, hybrid vesicle, drug delivery, intracellular uptake mechanism

## Abstract

Extracellular vesicles play an important role in intercellular communication, with the potential to serve as biomaterials for nanocarriers. Combining such extracellular vesicles and liposomes results in advanced drug delivery carriers. In this study, we attempted to fabricate hybrid vesicles using a membrane fusion method and incorporated an anticancer drug. As a result, we successfully prepared nanosized uniform hybrid vesicles and evaluated their physicochemical characteristics and intracellular uptake mechanisms via endocytosis in various cell lines. Compared to liposomes, the hybrid vesicles showed better physical properties and a relatively higher reduction in cell viability, which was presumably dependent on the specific cell type. These findings suggest that fusion-based hybrid vesicles offer a novel strategy for delivering therapeutic agents and provide insights into the types of extracellular vesicles that are useful in fabricating hybrid vesicles to develop an advanced drug delivery system.

## 1. Introduction

Extracellular vesicles (EVs) are small, membranous, lipid-bound structures that are produced by cells in the extracellular environment [1]. EVs, including microvesicles, exosomes, and apoptotic bodies, play crucial roles in intercellular communication [2]. Initially known as garbage disposal units, EVs have recently been recognized as signaling vehicles capable of delivering biological components, such as nucleic acids (e.g., miRNA), lipids, and proteins between cells [3]. Owing to their high transportability and cargo-targeting ability, EVs have been extensively studied as drug delivery carriers [4,5]. Their use offers several advantages, such as biocompatibility [6], low immunogenicity [7], and a cell-homing tendency [8]. However, EVs have limitations owing to the difficulty of loading additional drugs into their inner space [9]. The process of inward invagination from the endosomes during their natural formation makes it challenging to load therapeutic agents into EVs [10]. To overcome this drawback, electroporation has been attempted for loading therapeutic agents and improving their entrapment efficacy; however, it can induce damage, such as denaturation and aggregation of the vehicles during the process [11,12,13]. Other chemical-based transfection and simple incubation methods have also been reported [14]. Moreover, in practice, the use of EVs from the bench to in industry is hindered during large-scale production; their quality and productivity is influenced by the cell conditions and surroundings, resulting in inadequate quality control owing to batch-to-batch variance [15].

Recently, the fusion of exosomes and liposomes has emerged to address these limitations in practical applications [16]. Membrane fusion of lipid vesicles is achieved using various fabrication methods, such as freeze–thawing, incubation, and extrusion [16,17,18]. These approaches promise a high drug payload, high stability and biocompatibility, and low immunogenicity [19]. Based on the fabrication method employed, hybrid vesicles (HVs) that combine exosomes and liposomes exhibit different physicochemical properties. For example, HVs prepared by freeze–thawing suffer membrane damage, resulting in the potential leakage of therapeutic agents despite their high payloads [20]. HVs can also be prepared through natural incubation; although this process is simple, they show a low fusion efficacy [21]. Using polyethylene glycol-mediated fusion in the fabrication of hybrid vesicles enhanced the circulation time in the blood [22]. In contrast, HVs with stealth properties demonstrate reduced cellular uptake [23]. HVs prepared using the membrane extrusion method exhibited the following advantages—uniformity, a rapid fabrication process, and easy size controllability [24]—despite the potential shear stress during the extrusion process [25]. While these fabrication methods have showed encouraging proofs of concept for applications in antitumor therapy [26], the treatment of immunological diseases [27], and intracellular delivery [28], the detailed mechanisms of intracellular uptake and trafficking have not yet been fully elucidated. However, the cellular uptake of EVs has been reported to depend on their specificity [29,30]. To date, the specificity and recognition of EVs in recipient cells remain mostly unknown, and it is unclear whether artificially fabricated HVs containing EVs maintain these properties or not.

Herein, we aimed to fabricate HVs loaded with doxorubicin (DOX) and characterize them physiochemically. We delivered them to various types of cells to gain insight into the cellular uptake mechanisms. For a comprehensive understanding of intracellular drug delivery, we investigated the intracellular behavior of HVs and DOX inside the cells.

## 2. Materials and Methods

### 2.1. Materials

The doxorubicin hydrochloride was purchased from BLD Pharmatech (#BD32885, Shanghai, China). The l-α-phosphatidylcholine (#441601, Soy PC), Avanti^®^ Mini Extruder (#610000), and 1-oleoyl-2-[12[7-nitro-2-1,3-benzoxadiazol-4-yl]amino]-sn-glycero-3-phophocholine (#810133C, NBD-PC) were obtained from Avanti Polar Lipid Inc. (Alabaster, AL, USA). Dulbecco’s modified Eagle’s medium (#10-013-CV, DMEM), fetal bovine serum (#35-015-CV, FBS), and penicillin–streptomycin (#30-002-Cl) were purchased from Corning (Corning, VA, USA). Dulbecco’s Phosphate Buffered Saline (#14200075, DPBS 10X) was purchased from Gibco (Waltham, MA, USA), while the Cell Counting Kit-8 (CCK-8), a colorimetric assay for measuring water-soluble tetrazolium salt, was acquired from DoGenBio (#EZ-3000, EZ-Cytox, Seoul, Republic of Korea). The bicinchoninic acid assay kits (#23225, Pierce™ BCA Protein Assay Kits), LysoTracker^TM^ Deep Red (#L12492), and 4′,6-diamidino-2-phenylindole (#F6057, DAPI) were purchased from Thermo Scientific Inc. (Waltham, MA, USA), Invitrogen (Waltham, MA, USA), and Sigma-Aldrich (Fluoroshield^TM^ with DAPI St. Louis, MO, USA), respectively. Ultrapure water was obtained from Welgene Inc. (#ML109-02, Gyeongsan, Republic of Korea). All the other reagents and chemicals used were of analytical grade.

### 2.2. In Vitro Cell Culture

Human breast cancer cell lines, including MCF-7, MDA-MB-231, and MDA-MB-468, were used in this study. The MCF-7 (KCLB No. 30022) cell line was purchased from the Korean Cell Line Bank (Seoul, Korea), while MDA-MB-231 (ATCC^®^ No. HTB-26^TM^) and MDA-MB-468 (ATCC^®^ No. HTB-132^TM^) were obtained from Prof. H. Kim. Each cell line was grown in DMEM supplemented with 10% (*v*/*v*) FBS and 1% (*v*/*v*) penicillin–streptomycin. All the cells were cultured within a humidified 5% CO_2_ incubator at 37 °C.

### 2.3. Isolation of the Extracellular Vesicle

For EV harvesting, the MCF-7 cells were cultured in serum-free DMEM without phenol red for 48 h within a humidified 5% CO_2_ incubator at 37 °C, and each conditioned medium was collected. Prior to isolating the MCF-7-produced EVs, each supernatant was centrifuged at 2000× *g* for 30 min to remove unnecessary cell debris. Then, each of the conditioned media was concentrated using a centrifugal filter (Amicon^®^ Ultra-15 Centrifugal Filters, Millipore, MA, USA) through centrifugation at 3000× *g* at 4 °C for 15 min. The resulting EV-containing pellets were resuspended and sequentially extruded through a set of polycarbonate membranes in order of size with 0.4, 0.2, and 0.1 μm pores (PC Membranes, Avanti^®^ Polar Lipids Inc., Alabaster, AL, USA) using a Mini-Extruder (Avanti^®^ Polar Lipid). Each extrusion was repeated five times at each step, and then sequentially performed in the order of large to small pore sizes. The purified EVs were stored at −70 °C for further experiments [31,32,33,34].

The purified EVs were quantified using the BCA assay, which measures the total amount of protein in the EVs. Briefly, a set of protein standards was prepared with a linear range of 31.25–2000 μg/mL. The EVs were exposed to the working reagent, incubated at 37 °C for 30 min, and then detected using a multi-mode microplate reader (Synergy™ HTX Multi-Mode Microplate Reader, BioTek Instruments, Winooski, VT, USA) at 562 nm. These data were represented as mean ± standard deviation (*n* = 3) [24].

### 2.4. Preparation of the Liposomes

The liposomes were prepared using the thin-film hydration method. In brief, 10 mg of soy PC dissolved in chloroform was slowly evaporated in a rotary evaporator at 42 °C (Yamato Scientific Co., Ltd., Tokyo, Japan). The lipid thin film was flushed with nitrogen gas to completely remove all traces of the residual organic solvent. The dried lipid film was then hydrated, vigorously vortexed, and sonicated in a water bath for 10 min. To prepare the DOX-loaded liposomes, DOX was added during the hydration process. The prepared liposomes with/without DOX were sequentially extruded for five cycles through a polycarbonate membrane with a 0.4, 0.2, and 0.1 μm pore size in order using the mini-extruder. The prepared liposomes of uniform sizes were stored at 4 °C for further studies [32,35].

### 2.5. Preparation of the HVs

To label phosphatidyl choline, NBD-PC was used at a final concentration of 1 mol% [36]. The EV-containing aqueous solutions with/without DOX were incorporated during hydration to combine the EVs and liposomes. The lipid thin film, composed of phosphatidylcholine, was hydrated, vigorously vortexed, and sonicated in a water bath for 10 min, mimicking the thin-film hydration method. In this step, 50 μg/mL of DOX was added together and entrapped. HVs consisting of liposomes and EVs were fabricated at different weight ratios and further homogenized using the membrane extrusion method. The prepared HVs with/without DOX were sequentially extruded for five cycles through a polycarbonate membrane with a 0.4, 0.2, and 0.1 μm pore size in order using the mini-extruder [34,37].

### 2.6. Physicochemical Properties of the Lipid Vesicles

#### 2.6.1. Size and Zeta Potential Values of the Lipid Vesicles

The size, polydispersity index (PDI), and zeta potential values were measured using the Zetasizer Nano ZS90 (Malvern Panalytical, Worcestershire, UK) at 25 °C. The data were measured in triplicate, represented as mean ± standard deviation (*n* = 3).

#### 2.6.2. Cryo-TEM Analysis of the Lipid Vesicles

To visualize the HVs, cryo-transmission electron microscopy (cryo-TEM) was used to investigate the fusion of the exosomes and liposomes. The structures of each sample were characterized using Cryo-TEM (FEI Tecnai F20 G2, FEI, Hillsboro, OR, USA). Briefly, 3 μL of each sample solution was dropped on top of a holey carbon grid at 24 °C and saturated in terms of humidity. After dropping the solution, the grid was promptly blotted for 2 s and plunge-frozen below −170 °C into the precooled liquid ethane using Vitrobot (Vitrobot Mark IV, FEI, Hillsboro, OR, USA). The samples were analyzed using Cryo-EM at 200 kV.

### 2.7. Quantification of DOX

Free DOX (not entrapped) was removed through ultracentrifuging at 150,000× *g* at 4 °C for 3 h (Optima L-100XP, Beckman Coulter, Inc., Brea, CA, USA). The pellets were dissolved in 1 mL of 1% Triton-X for vesicle lysis [38,39]. The entrapment efficiency of DOX was quantitatively analyzed using high-performance liquid chromatography (HPLC, Agilent 1100 series, Agilent Technologies, Santa Clara, CA, USA). A C_18_ reverse-phase HPLC column (Supersil 120 ODS II, 4.6 × 150 mm, 5 μm particles, LB science, Seoul, Republic of Korea) was employed. The mobile phase consisted of an aqueous phase containing 25 mM monobasic potassium phosphate buffer, with methanol as the organic phase. An isocratic mobile phase comprising aqueous buffer and organic solvent was mixed at a ratio of 37:63 (*v*/*v*) and pH-adjusted to 2.8. The isocratic mobile phase was used for elution at a flow rate of 1 mL/min, and the injection volume was 20 μL. Standard concentrations of DOX were dissolved in the mobile phase, and each eluent was monitored at 254 nm [40].

### 2.8. Physical Stability of the Lipid Vesicles

The storage stability of the vesicles was assessed at 4 °C in saline and at 37 °C in the cell culture medium (DMEM supplemented with 10% FBS). Briefly, each pellet was resuspended in saline or cell culture medium after ultracentrifugation, as described above. The colloidal stability of both the liposomes and HVs was examined under the given conditions using the Zetasizer (Malvern Panalytical). Both the size and PDI of the vesicles were monitored at scheduled time intervals in saline or the cell culture medium.

The physical stability of these vesicles was evaluated following Triton X-100 treatment, a representative destabilizer. In brief, 1 mL of each formulation was adjusted to 0.16 mM, 0.64 mM, 0.96 mM, and 1.6 mM of Triton X-100. Changes in size and PDI were measured using the Zetasizer. All the data were measured in triplicate, represented as mean ± standard deviation (*n* = 3) [41,42,43].

### 2.9. In Vitro Cell Viability of the Lipid Vesicles

The cells (1 × 10^4^ cells/well) in 96-well plates were pre-seeded and incubated at 37 °C for 1 day. Upon adherence, the formulations without DOX were treated for 24 h, whereas the formulations with DOX were treated for 4 h. Subsequently, DMEM containing 10% (*v*/*v*) CCK-8 reagent, a colorimetric assay for determining the cell viability, was added to each well and further incubated at 37 °C. The absorbance was measured at 450 nm using a multimode microplate reader, and the relative cell viability was normalized to that of the control group without formulations. The data were shown as mean ± standard deviation (*n* = 4) [37,44,45].

### 2.10. Measurement of the Cellular Uptake of the Lipid Vesicles

The cellular uptake of both the HVs and liposomes was evaluated using confocal laser scanning microscopy (Carl Zeiss LSM800, Zeiss, Jena, Germany). For confocal observation, each cell type was seeded onto cover glasses (Marienfeld GmbH & Co., Lauda-Königshofen, Germany) in a 6-well plate coated with 0.01% poly-*L*-lysine (PLL solution, Sigma). After 24 h of incubation, these cells were exposed to DMEM containing the HVs or liposomes and further incubated for 4 h. During incubation, 100 nM of LysoTracker (LysoTracker^TM^ Deep Red, Invitrogen) was added for 2 h to label both the late and early lysosomes. Subsequently, the cover glasses were washed twice with PBS and stained with DAPI for 30 min at 25 °C before microscopic observation. Images were acquired using confocal microscopy. The optimal conditions for microscopic observation were determined according to the following conditions [35,45]: DAPI (laser illumination wavelength and power: 405 nm and 10.0%; excitation at 353 nm, emission at 465 nm; detection range: 400–500 nm), DOX (laser illumination wavelength and power: 488 nm and 12.0%; excitation at 501 nm, emission at 552 nm; detection range: 580–617 nm), LysoTracker (laser illumination wavelength and power: 640 nm and 10.0%; excitation at 641 nm, emission at 662 nm; detection range: 656–700 nm), and NBD-PC (laser illumination wavelength and power: 488 nm and 12.0%; excitation at 459 nm, emission at 529 nm; detection range: 410–540 nm), which were observed at ×63.

### 2.11. Cellular Uptake Mechanism of the Lipid Vesicles

To investigate the cellular uptake mechanisms, various endocytic inhibitors were used, according to previous studies [46]. The cellular uptake of the vehicles was relatively quantified using a flow cytometry system (BD FACSCalibur™, BD Biosciences, San Jose, CA, USA). The cells were pre-seeded in a 6-well plate for 24 h. The cells were then washed with DPBS and preincubated with the following endocytic inhibitor at 37 °C for 1 h before liposome or HV treatment: cytochalasin D (CytD, 10 µg/mL), methyl-β-cyclodextrin (MβCD, 5 mg/mL), wortmannin (WMN, 100 ng/mL), pertussis toxin (PTX, 100 ng/mL), chlorpromazine (CPM, 10 µg/mL), or dynasore (80 µM) [47]. After treatment, the cells were again washed with DPBS and trypsinized with 0.05% Trypsin in 0.53 mM EDTA (Corning, VA, USA). After harvesting the trypsinized cells, the supernatant was removed through centrifugation, and the remaining cells were fixed with 4% paraformaldehyde for 30 min at 25 °C. The cell suspension was centrifuged and resuspended with 500 μL of cold DPBS. Untreated cells were used as the control, and 10,000 cells were counted per event. The fluorescence intensity of DOX was measured using a FL2-H filter. The relative fluorescence intensity was analyzed using FlowJo^TM^ v10.10 software (TreeStar, Inc., Ashland, OR, USA) according to the gating strategy in the flow cytometry system. The mean fluorescence intensity (MFI) was calculated, and all the values were normalized to the control cells treated with each formulation. The measurement conditions were indicated by the following conditions: illumination wavelength (488 nm), detector (FL2, filter; 585/42), and power in the MCF-7 cell (430 voltage), the MDA-MB-231 cell (420 voltage), and the MDA-MB-468 cell (383 voltage). These data were measured as mean ± standard deviation (*n* = 3) [32,47].

### 2.12. Statistics

All the comparisons between the two groups were tested using a one-tailed unpaired Student’s *t*-test. One-way ANOVA analysis with post hoc Bonferroni correction was used to compare means among more than two groups. Differences were considered statistically significant when * *p* < 0.05, ** *p* < 0.01, and *** *p* < 0.001.

## 3. Results

### 3.1. Physicochemical Characterization of the HVs

Prior to fabricating the HVs, EVs isolated from the MCF-7 cells were characterized using dynamic light scattering and zeta potential analysis. The EVs were quantitatively analyzed using the BCA assay. As shown in Table S1, the size and zeta potential value of the EVs were 189.53 ± 7.27 nm and −18.33 ± 0.52 mV, respectively. The prepared EVs approximately 200 nm in size were uniformly formed, which included the proteins within the vesicles. In the EVs, the total amount of proteins was 20.87 ± 0.01 μg, which were obtained from 5.5 × 10^7^ MCF-7 cells. The isolated EVs were well characterized, and their amounts were quantitatively analyzed to prepare the HVs through the fusion process.

**Figure 1:**
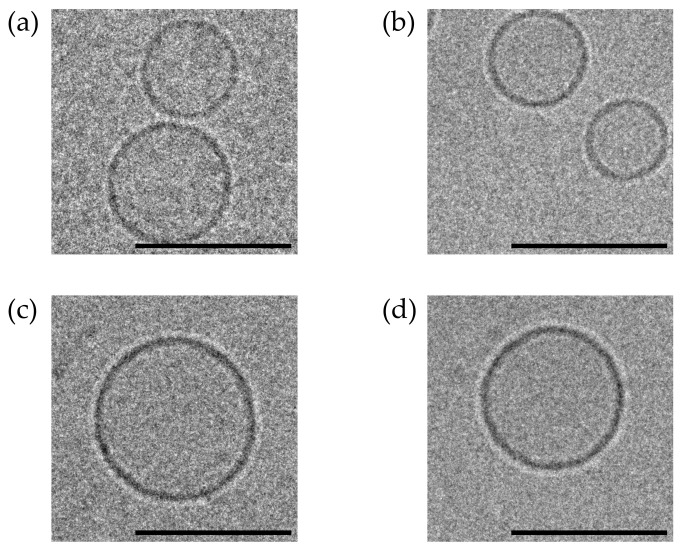
Cryo-TEM images of HVs. (**a**) F1, (**b**) F2, (**c**) F3, (**d**) F4. Scale bars were represented by 100 nm.

Various HVs depending on the weight ratios were fabricated, as described in Materials and Methods. The physicochemical properties of the HVs without DOX were investigated, as shown in Table 1. The liposomes were weighed depending on the amount of phosphatidylcholine, whereas the EVs were weighed based on the amount of proteins, which was quantified using the BCA assay. As a result, the HVs were less than 150 nm in size, slightly negatively charged, and monodispersed. The PDI value decreased after the serial extrusion process; however, no significant differences were observed between the liposomes and HVs. Cryo-transmission electron microscopy images confirmed the spherical shape of the vesicles, as shown in Figure 1. The fabricated HVs showed a morphology similar to that of the liposomes, a uniform size below 130 nm, and a slightly negative surface charge. Based on these data, it was confirmed that the HVs were successfully fabricated through the fusion process. To investigate the use of HVs as drug delivery vehicles, DOX, a representative anticancer drug, was loaded into each formulation. The DOX-loaded liposomes and HVs were physicochemically characterized in terms of their size, zeta potential value, and polydispersity. As a result, DOX was entrapped inside the vesicles; however, the amount of DOX was not significantly affected by the formulation, as shown in Table S2 (*** *p* < 0.001). Despite DOX loading, the HVs showed similar sizes and maintained low PDI values compared to the HVs without DOX; see Table 1. These DOX-loaded vesicles were subsequently used for the cellular uptake studies and compared with each other depending on the route of cellular uptake in vitro.

### 3.2. Physical Stability of the Lipid Vesicles

Lipid-based vesicles have some limitations related to their stability, despite being safe, biodegradable, and well established compared to other delivery vehicles. Hence, the enhanced stability of lipid-based vesicles is beneficial under physiological environments. To investigate the stability of the HVs, the following aspects were evaluated and compared with those of the liposomes.

**Figure 2:**
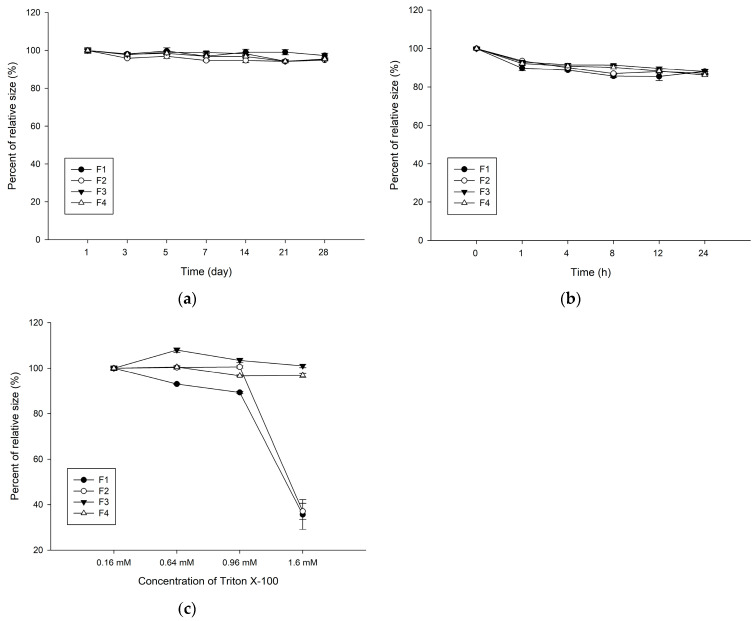
Physical stability of vesicles from F1 to F4. Relative changes in size were measured in (**a**) saline, (**b**) 10% FBS, and (**c**) Triton X-100.

The physical stability of the HVs was evaluated in saline and 10% FBS. As mentioned in the Materials and Methods section, each vesicle, from F1 to F4, was stored under the following conditions. First, each vesicle was stored at 4 °C for 4 weeks, as shown in Figure 2a. None of the vesicles showed significant differences in size. Next, in Figure 2b, each vesicle was stored in 10% FBS and monitored at 37 °C for 24 h. In the early stage (within 1 h), each vesicle size was slightly reduced by approximately 10%. However, each vesicle was maintained and showed no significant difference between the liposomes and HVs. Additional stability studies were performed in the presence of surfactants such as Triton X-100, a representative non-ionic surfactant that disrupts vesicles and solubilizes aggregates of biologics [48]. As shown in Figure 2c, each vesicle was resuspended in various concentrations of Triton X-100 (0.16–1.6 mM). All the vesicles showed no considerable changes below 0.96 mM; however, sizes of F1 and F2 were not detected at 1.6 mM, indicating that most of the vesicles were disrupted. F2 was not stable at 1.6 mM of Triton X-100, resulting in the less stable F2 being excluded and not tested in further experiments. Taken together, all the vesicles from F1 to F4 were stable in saline and 10% FBS, whereas the HVs (F3 and F4) were stable at relatively high concentrations of Triton X-100.

### 3.3. In Vitro Cytotoxicity of the DOX-Loaded Lipid Vesicles

Prior to the anticancer effect of the HVs, the fabricated HVs without DOX were measured in terms of the cytotoxicity of the vehicles, which was quantitatively analyzed using the CCK-8 assay. As shown in Figure S1, none of the HVs (F3–F4) or liposomes (F1) showed significant cytotoxicity in the MCF-7, MDA-MB-231, and MDA-MB-468 cells, indicating their applicability as drug delivery carriers (* *p* < 0.05, ** *p* < 0.01, and *** *p* < 0.001).

**Figure 3:**
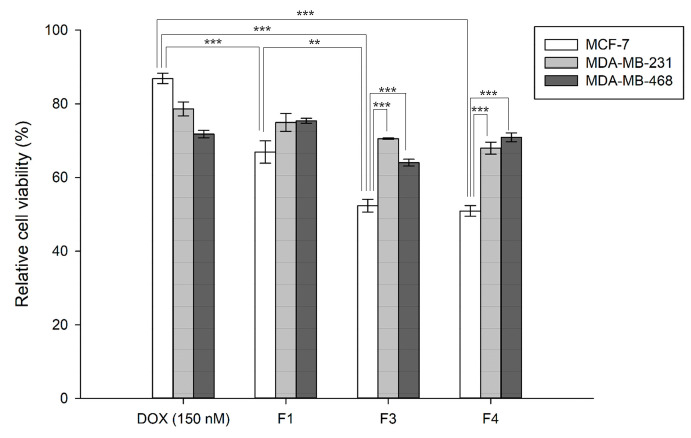
Cell cytotoxicity of HVs with DOX in vitro. MCF-7, MDA-MB-231 and MDA-MB-468 cells were treated 150 nM DOX and HVs for 4 h. Relative cell viability was normalized to the control group in each cell line, respectively. (** *p* < 0.01, and *** *p* < 0.001).

To evaluate the cellular uptake and concomitant cytotoxicity, DOX was applied to various mammary cancer cell lines. Compared with DOX without the use of vehicles, the lipid-based vehicles showed a better cytotoxic effect, as shown in Figure 3. DOX (150 nM) reduced the cell viability by approximately 13.11%, whereas the DOX-loaded liposomes (F1) exhibited 33.09% cytotoxicity in the MCF-7 cells. Interestingly, the DOX-loaded HVs fabricated with the MCF-7-originating EVs showed a higher cytotoxic effect on the MCF-7 cells as follows: F3 (47.64%) and F4 (49.08%). Compared to DOX alone, the liposomes (F1) increased the cytotoxic effect by more than 2.5 times and the HVs by more than 3.5 times (*** *p* < 0.001). In the MDA-MB-231 cells, F1 showed an inhibitory effect of approximately 25.06%, whereas F3 and F4 showed 29.48% and 32.03%, respectively. These HVs were less effective against the MDA-MB-231 cells, although lipid-based vesicles showed better cytotoxic effects than DOX alone. In the MDA-MB-468 cells, the HVs exhibited reduced viability as follows: F3 (35.94%) and F4 (29.09%). These HVs showed better cytotoxic effects than F1 (liposomes) and DOX alone. These findings demonstrate that lipid-based vesicles are effective and beneficial for the intracellular delivery of anticancer agents. More importantly, this suggests that HVs respond differently, depending on the cell type. Before delving into the detailed mechanisms of cellular uptake, we investigated whether such cellular uptake was ATP-dependent, as shown in Figure S2 (* *p* < 0.05, ** *p* < 0.01, and *** *p* < 0.001). The results indicated that the cell viability was less reduced at 4 °C than at 37 °C, demonstrating that the cellular uptake of the lipid vesicles was partially ATP-dependent compared to the physiological growth conditions. At 4 °C, in terms of energy depletion, the lipid-based vesicles showed a lower cellular uptake, indicating an endocytosis pathway involving a general energy-consuming process, with no significant differences among formulations. In addition, no significant difference was observed between the HVs (F3, F4), whereas the inhibition of cell viability showed some differences among F1, F3, and F4 (*** *p* < 0.001); F3 and F4 demonstrated a better inhibition effect than the liposomes (F1), as observed in each type of cell. These data suggest that HVs are more effective and exhibit a better inhibitory effect on cancer cell viability after treatment with DOX-loaded vesicles.

### 3.4. Cellular Uptake Mechanisms of the DOX-Loaded Vesicles

**Figure 4:**
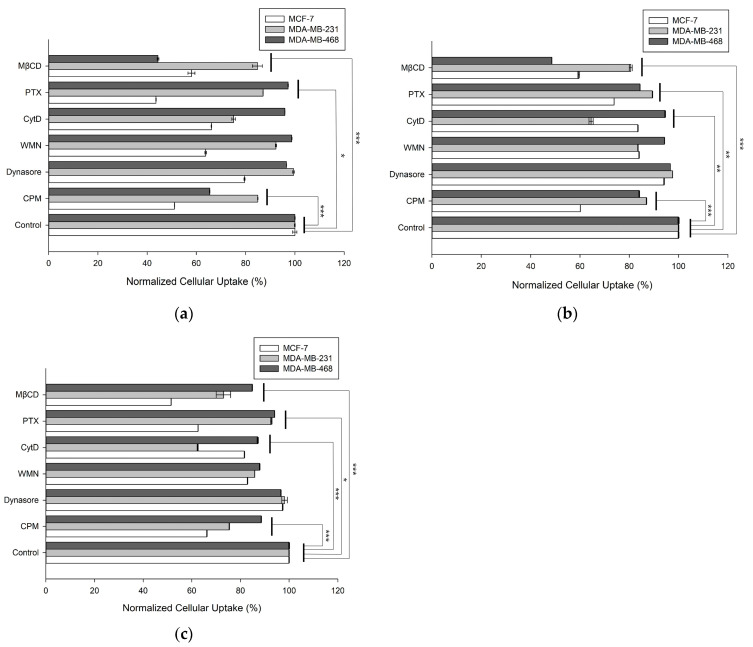
Normalized intracellular uptake of (**a**) F1, (**b**) F3, and (**c**) F4 in MCF-7, MDA-MB-231, and MDA-MB-468 cells when co-incubated with inhibitors such as MβCD, PTX, CytD, WMN, dynasore, and CPM. (* *p* < 0.05, ** *p* < 0.01, and *** *p* < 0.001).

To better understand the cellular uptake mechanisms, several endocytic inhibitors were used during the internalization process. To determine which endocytic pathway was responsible for cellular uptake, the cells were pretreated with the following inhibitors for 1 h, and then each vesicle with DOX was co-incubated in each cell type: CPM for clathrin-mediated endocytosis, MβCD for caveolae-mediated endocytosis, CytD for clathrin/caveolae-dependent endocytosis, WMN for macropinocytosis, PTX for G protein-coupled receptor-mediated endocytosis, and dynasore for dynamin-dependent endocytosis. As shown in Figure 4a, F1 cellular uptake in the MCF-7 cells was primarily inhibited by CPM, WMN, CytD, PTX, and MβCD, which are involved in clathrin-mediated endocytosis, macropinocytosis, clathrin/caveolae-dependent endocytosis, G protein-coupled receptor-mediated endocytosis, and caveolae-mediated endocytosis, respectively. In contrast, the cellular uptake of F1 occurred via various routes in the MDA-MB-231 cells and mainly through caveolae-dependent and clathrin-dependent endocytosis in the MDA-MB-468 cells. In Figure 4b,c, F3 and F4 were taken up into the MCF-7 cells through caveolae-mediated, G-protein coupled receptor-mediated, and clathrin-mediated endocytosis, resulting from inhibition by CPM, PTX, and MβCD, respectively. Conversely, F3 was internalized via clathrin/caveolae-dependent endocytosis in the MDA-MB-231 and MDA-MB-468 cells. F4 was taken up by the MDA-MB-231 cells via clathrin/caveolae-mediated endocytosis; however, no critical route of cellular uptake was noted in the MDA-MB-468 cells. Based on these data, F1, F3, and F4 showed subtle differences depending on the composition of the HVs and cell types (* *p* < 0.05, ** *p* < 0.01, and *** *p* < 0.001). In addition, we confirmed whether the lipid vesicles, such as the liposomes and HVs, effectively delivered DOX into the cells, indicating that the lipid vesicles were appropriate for delivering DOX. The lipid vesicles, including liposomes and HVs, were predominantly endocytosed, which was confirmed using overlapping images of the lipid vesicles stained with NBD-PC (green fluorescence) and late endosome/early lysosomes stained with LysoTracker (Deep Red fluorescence) (Figure S3). After cellular uptake, DOX was also distributed inside the cells and then intercalated with the double-stranded DNA of the cells, evident from the overlapping of the nuclei stained using DAPI (blue fluorescence) with DOX (red fluorescence). These findings were additionally supported by histogram analysis using flow cytometry (Figure S4). Based on these data, the HVs were delivered into cells via energy-dependent and energy-independent endocytic pathways, depending on the EV-derived components of the HVs and recipient cells.

## 4. Discussion

EVs represent promising nanovesicles composed of lipid bilayers suitable for therapeutic agents [49]. Their advantages, such as target specificity, physical stability, biocompatibility, stimuli-responsiveness, and low immunogenicity, favors them as carriers for therapeutic agents [50]. However, obstacles persist, notably in large-scale production and drug loading [51]. Hence, we attempted to fabricate HVs combining liposomes and EVs and loaded them with DOX using an extrusion method with various fusion strategies [52]. Prior to the preparation of the HVs, we hypothesized that an optimal ratio of liposomes to EVs would influence the intracellular uptake. None of the vesicles from F1 and F4 showed significant differences and were similar when observed using cryo-TEM. All the vesicles retained their storage stability in a physiological solution and culture medium, indicating their colloidal stability and safety during in vitro cell studies. However, an increased Triton X-100 concentration compromised the colloidal stability of F1 and F2 at 1.6 mM. The liposomes start to solubilize over 1 mM [48,53], which is sufficient to disrupt vesicles. Interestingly, F3 and F4, with a higher EV composition, maintained their structural stability, implying the importance of the EV quantity to membrane fusion and HV stability. In this regard, we presume that the improved stability of the HVs is attributed to the rigidity of lipid vesicles with biological components of EVs.

DOX is a well-established chemotherapeutic agent that is clinically used to treat various types of cancers, including mammary, lung, glioblastoma, and thyroid cancers [54]. DOX exhibits intrinsic fluorescence, which is useful for monitoring cellular uptake [55]. Hence, we used DOX in this study and attempted to confirm how lipid vesicles are effectively delivered and taken up inside the cells, depending on their composition and/or cell type. Liposomes and EVs have been shown to internalize via different routes [56], suggesting that the internalization mechanisms of our HVs into cells could be placed between those of liposomes and EVs. Based on our results, the DOX-loaded lipid vesicles were predominantly delivered through endocytosis and reached the DNA in the nucleus, as observed using confocal microscopy. The confocal images of DAPI and DOX overlapped, indicating intercalation with DNA, leading to cytotoxicity [57]. DOX when used without the vesicles was purportedly internalized via passive diffusion across the membrane owing to its small molecular weight (Mw = 543.52 g/mol) and weak basic nature (pKa = 8.3) [58], whereas the DOX-lipid vesicles were endocytosed according to our data. In particular, F1, F3, and F4 were endocytosed but showed subtle differences during internalization. These variations are likely attributed to biological factors, such as the insertion of biomolecules, as well as physicochemical factors, such as changes in the rigidity of the lipid vesicles. Overall, we believe that such a relatively small difference in the lipid vesicles influences their physical properties, cellular uptake, and concomitant therapeutic effects, implying that various factors should be considered in the design of fusion vesicles like HVs.

The fabricated HVs have a structure resembling that of liposomes or EVs and show superior physical properties, good biocompatibility, and an improved cellular uptake. Despite existing challenges in practical production and clinical translation, HVs could be alternatives as future drug carriers. In future, multifunctional vesicles should be developed based on this approach, paving the way for personalized medicine using nano/bioconvergence.

## 5. Conclusions

In conclusion, stable HVs combining liposomes and EVs were well fabricated and exhibited an improved cellular uptake of DOX by the cancer cells. The internalization mechanisms of the HVs, contingent upon the composition of the HVs and cell types, aligned partially with ATP-dependent endocytosis. This report serves as a proof of concept for HV preparation using the fusion process, offering a promising perspective for future generations of drug delivery carriers.

## Figures and Tables

**Table 1:** Physicochemical properties of liposome and HVs.

Formulation	Composition ^1^	Size (nm)	Polydispersity Index (PDI)	Zeta Potential Value (ζ, mV)
F1	Liposome	134.90 ± 0.49	0.10 ± 0.01	−5.37 ± 0.24
F2	Lipo:Exo (10:1)	129.17 ± 0.45	0.08 ± 0.00	−6.16 ± 0.22
F3	Lipo:Exo (5:1)	119.03 ± 0.34	0.09 ± 0.01	−6.05 ± 0.35
F4	Lipo:Exo (2:1)	113.60 ± 0.52	0.11 ± 0.01	−9.75 ± 0.58

^1^ Weight ratio of composition in lipid vesicles (*w*:*w*).

## Data Availability

Data are available upon reasonable request to the corresponding author.

## Supplementary Materials

The following supporting information can be downloaded at https://www.mdpi.com/article/10.3390/pharmaceutics16040440/s1. Table S1: Size, polydispersity index, zeta potential values of EVs; Table S2: Physicochemical properties of liposomes and HVs with DOX; Figure S1: Cell viability of HVs without DOX in (a) MCF-7, (b) MDA-MB-231, (c) MDA-MB-468 cells for 24 h. (* *p* < 0.05, ** *p* < 0.01, and *** *p* < 0.001); Figure S2: Cell toxicity of HVs with DOX in MCF-7 for 4 h at 4 °C and 37 °C. (** *p* < 0.01, and *** *p* < 0.001); Figure S3a: Cellular uptake of F1, F3, and F4 in MCF-7. For trafficking of DOX (red)-loaded formulations, the following dyes were used: DAPI (blue) for nuclei, NBD-PC (green) for lipid vesicles such as liposomes and HVs, and LysoTracker (Deep Red) for late endosomes/early lysosomes. Scale bar is 10 μm. Colocalization was quantitatively analyzed using Pearson’s correlation coefficients using the Image J version 1.53e program between DAPI and DOX (DAPI-DOX), DAPI and Lysotracker (DAPI-Lyso), between DOX and NBD-PC (DOX-NBD-PC) and Lysotracker and NBD-PC (Lyso-NBD-PC), respectively. (* *p* < 0.05, ** *p* < 0.01, and *** *p* < 0.001); Figure S3b: Cellular uptake of F1, F3, and F4 in MDA-MB-231. For trafficking of DOX (red)-loaded formulations, the following dyes were used: DAPI (blue) for nuclei, NBD-PC (green) for lipid vesicles such as liposomes and HVs, and LysoTracker (Deep Red) for late endosomes/early lysosomes. Scale bar is 10 μm. Colocalization was quantitatively analyzed using Pearson’s correlation coefficients using the ImageJ version 1.53e program between DAPI and DOX (DAPI-DOX), DAPI and LysoTracker (DAPI-Lyso), DOX and NBD-PC (DOX-NBD-PC), and LysoTracker and NBD-PC (Lyso-NBD-PC), respectively. (* *p* < 0.05, and ** *p* < 0.01); Figure S3c: Cellular uptake of F1, F3, and F4 in MDA-MB-468. For trafficking of DOX (red)-loaded formulations, the following dyes were used: DAPI (blue) for nuclei, NBD-PC (green) for lipid vesicles such as liposomes and HVs, and LysoTracker (Deep Red) for late endosomes/early lysosomes. Scale bar is 10 μm. Colocalization was quantitatively analyzed using Pearson’s correlation coefficients using the ImageJ version 1.53e program between DAPI and DOX (DAPI-DOX), DAPI and LysoTracker (DAPI-Lyso), DOX and NBD-PC (DOX-NBD-PC), and LysoTracker and NBD-PC (Lyso-NBD-PC), respectively. (* *p* < 0.05); Figure S4a: Flow cytometry analysis for cellular uptake with endocytosis inhibitors of (a) F1, (b) F3, (c) F4 in MCF-7; Figure S4b: Flow cytometry analysis for cellular uptake with endocytosis inhibitors of (a) F1, (b) F3, (c) F4 in MDA-MB-231; Figure S4c: Flow cytometry analysis for cellular uptake with endocytosis inhibitors of (a) F1, (b) F3, (c) F4 in MDA-MB-468.
