# Supplementary material for: Engineered Hybrid Vesicles and Cellular Internalization in Mammary Cancer Cells
Source: Pharmaceutics. 2024 Mar 22;16(4):440. doi: 10.3390/pharmaceutics16040440 (PMC11054022; doi:10.3390/pharmaceutics16040440)
Supplement: Supplementary file 1 [file pharmaceutics-16-00440-s001.zip › pharmaceutics-2854951-supplementary.pdf]

Supplementary Table S1. **Size, polydispersity index, zeta potential values of EVs**

	Size (nm)	Polydispersity index (PDI)	Zeta potential value (ζ, mV)	Protein amount (μg)
**EVs**	189.53 ± 7.27	0.37 ± 0.02	-18.33 ± 0.52	20.87 ± 0.01

Supplementary Table S2. Physicochemical properties of liposome and HVs with DOX

Formulation	Formulation of Composition[1]	Size (nm)	Polydispersity index (PDI)	Zeta potential value (ζ, mV)
**F1**	Liposome	130.73 ± 0.41	0.10 ± 0.00	-2.91 ± 0.11
**F2**	Lipo:EV (10:1)	133.6 ± 0.31	0.10 ± 0.01	-2.92 ± 0.18
**F3**	Lipo:EV (5:1)	120.53 ± 0.55	0.13 ± 0.01	-6.23 ± 0.36
**F4**	Lipo:EV (2:1)	123.57 ± 0.38	0.14 ± 0.00	-8.15 ± 0.05

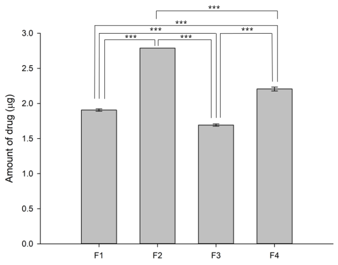

[1]Weight ratio of composition in vesicles (w:w), $^{***}p < 0.001$

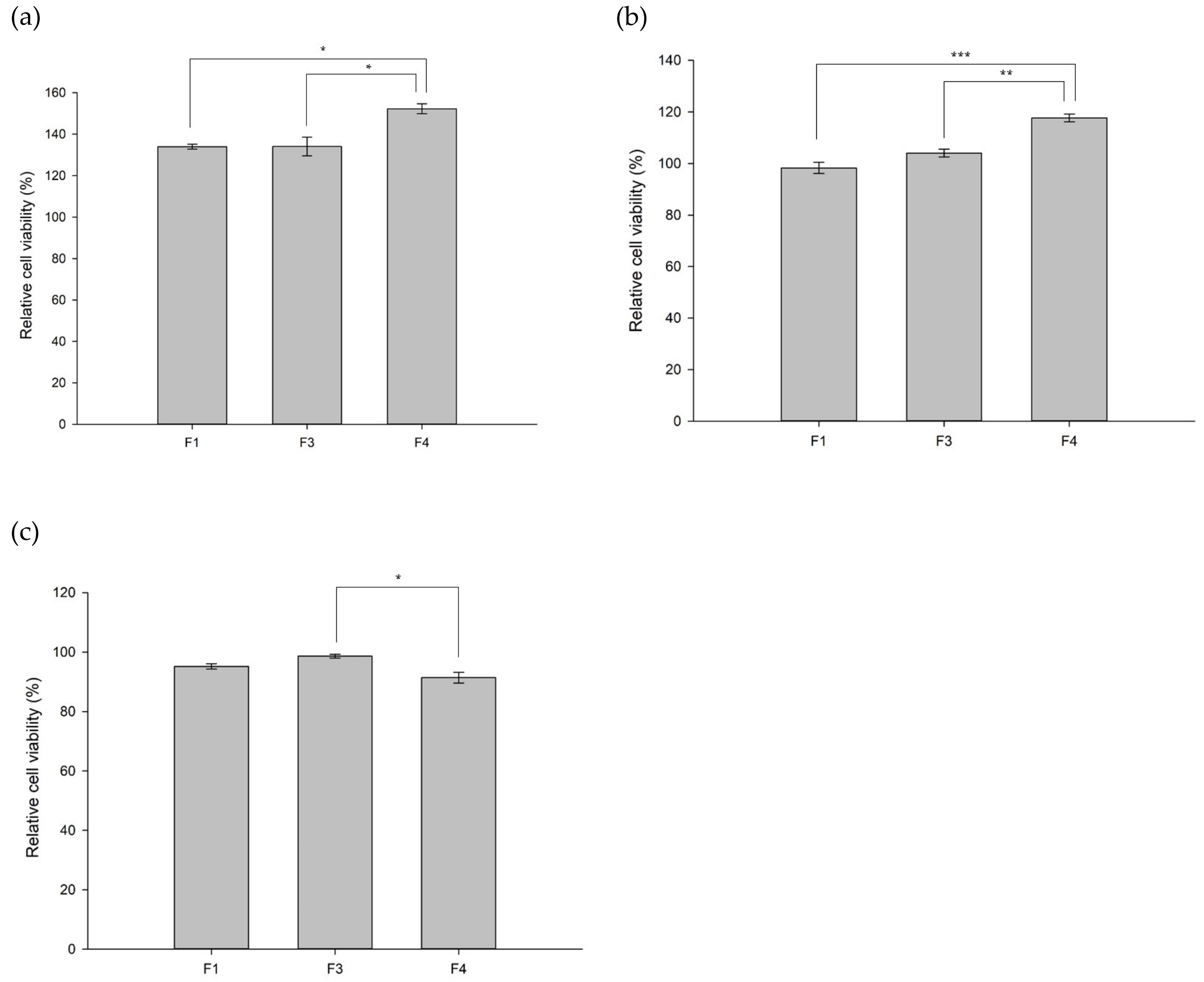

Figure S1. Cell viability of HVs without DOX in (a) MCF-7, (b) MDA-MB-231, (c) MDA-MB-468 cells for 24 h. ($^{*}p < 0.05$, $^{**}p < 0.01$, and $^{***}p < 0.001$)

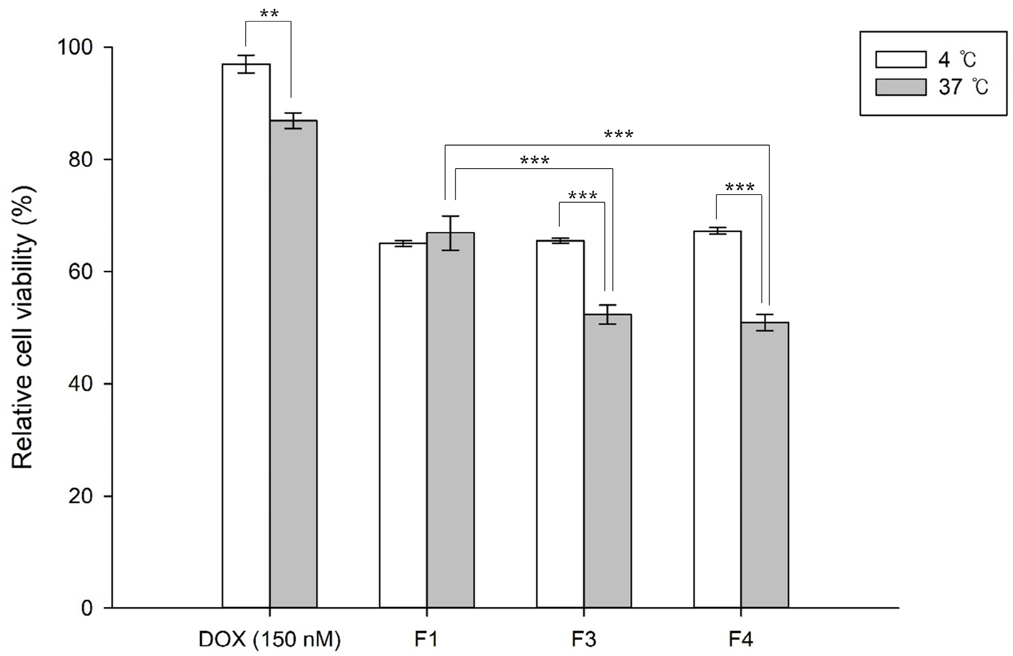

Figure S2. Cell toxicity of HVs with DOX in MCF-7 for 4 h at 4°C and 37°C. ($^{**}p < 0.01$, and $^{***}p < 0.001$)

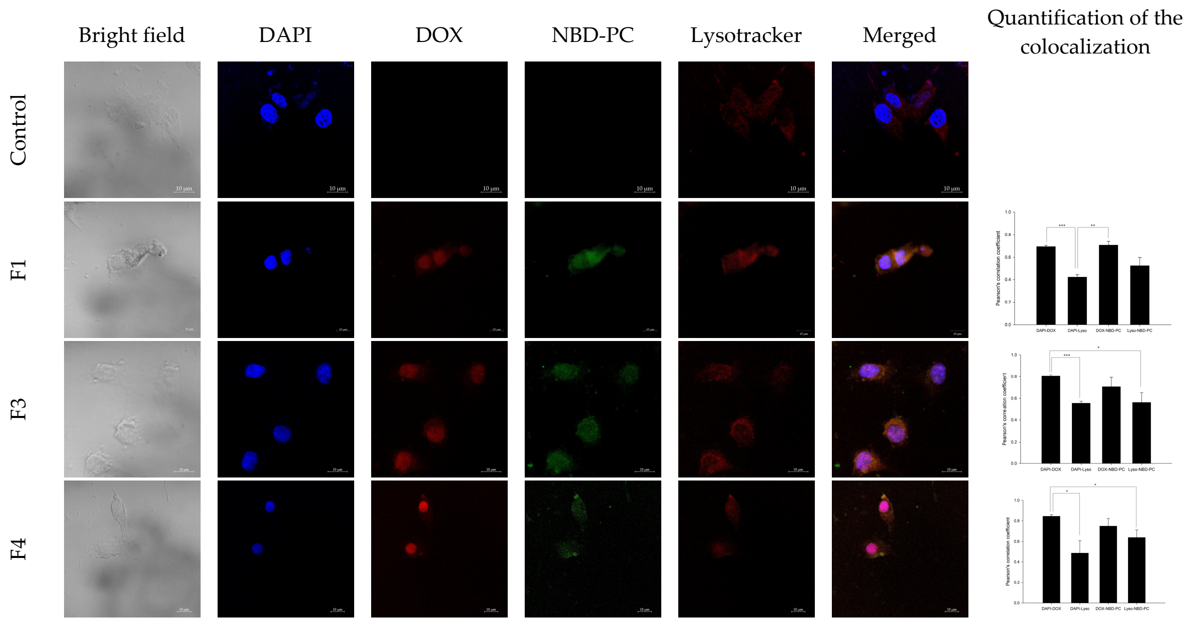

Figure S3a. Cellular uptake of F1, F3, and F4 in MCF-7. For trafficking of DOX (red)-loaded formulations, the following dyes were used: DAPI (blue) for nuclei, NBD-PC (green) for lipid vesicles such as liposomes and HVs, and LysoTracker (Deep Red) for late endosomes/early lysosomes. Scale bar is 10 μm. Colocalization was quantitatively analyzed using Pearson's correlation coefficients using the Image J version 1.53e program between DAPI and DOX (DAPI-DOX), DAPI and Lysotracker (DAPI-Lyso), between DOX and NBD-PC (DOX-NBD-PC) and Lysotracker and NBD-PC (Lyso-NBD-PC), respectively. (* $p < 0.05$, ** $p < 0.01$, and *** $p < 0.001$).

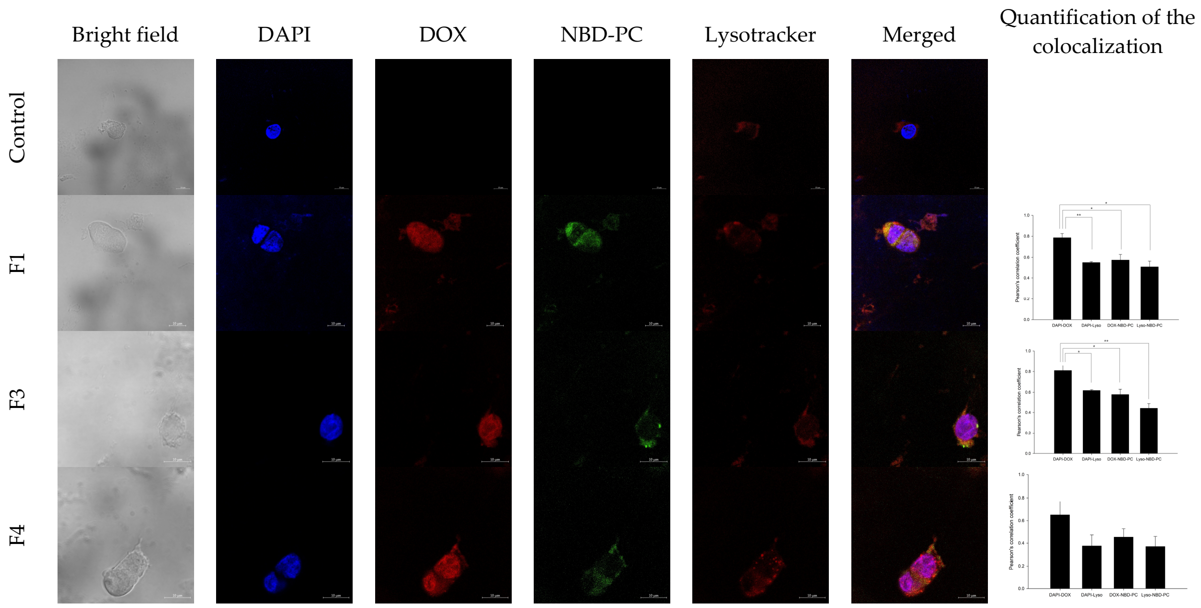

Figure S3b. Cellular uptake of F1, F3, and F4 in MDA-MB-231. For trafficking of DOX (red)-loaded formulations, the following dyes were used: DAPI (blue) for nuclei, NBD-PC (green) for lipid vesicles such as liposomes and HVs, and LysoTracker (Deep Red) for late endosomes/early lysosomes. Scale bar is 10 μm. Colocalization was quantitatively analyzed using Pearson's correlation coefficients using the ImageJ version 1.53e program between DAPI and DOX (DAPI-DOX), DAPI and LysoTracker (DAPI-Lyso), DOX and NBD-PC (DOX-NBD-PC), and LysoTracker and NBD-PC (Lyso-NBD-PC), respectively. (* $p < 0.05$, and ** $p < 0.01$).

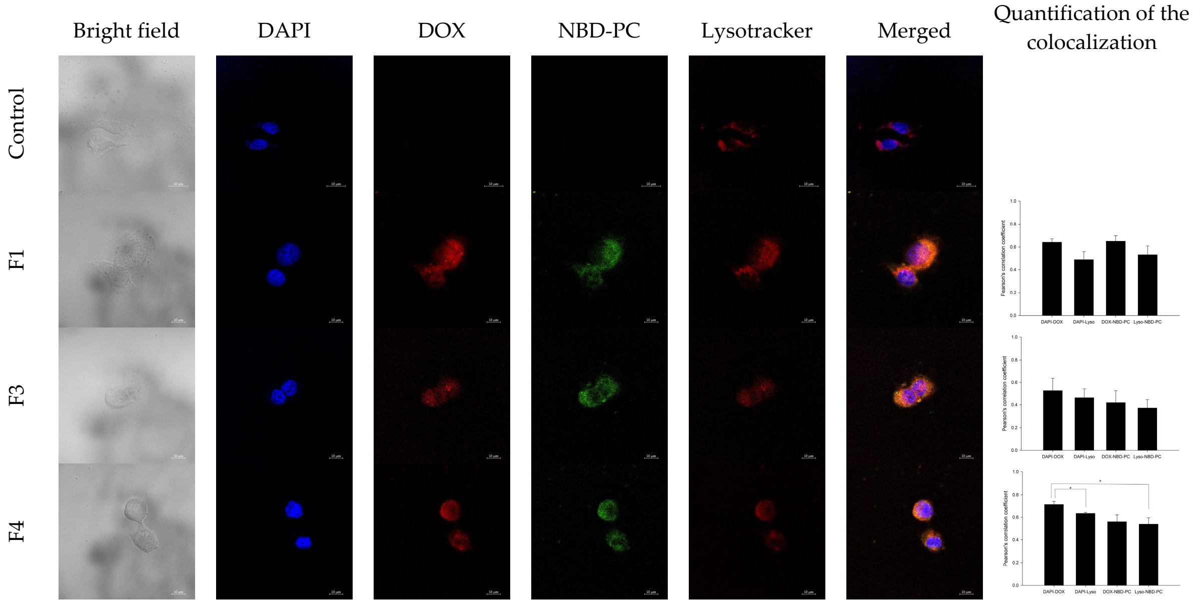

Figure S3c. Cellular uptake of F1, F3, and F4 in MDA-MB-468. For trafficking of DOX (red)-loaded formulations, the following dyes were used: DAPI (blue) for nuclei, NBD-PC (green) for lipid vesicles such as liposomes and HVs, and LysoTracker (Deep Red) for late endosomes/early lysosomes. Scale bar is 10 μm. Colocalization was quantitatively analyzed using Pearson's correlation coefficients using the ImageJ version 1.53e program between DAPI and DOX (DAPI-DOX), DAPI and LysoTracker (DAPI-Lyso), DOX and NBD-PC (DOX-NBD-PC), and LysoTracker and NBD-PC (Lyso-NBD-PC), respectively. (* $p < 0.05$).

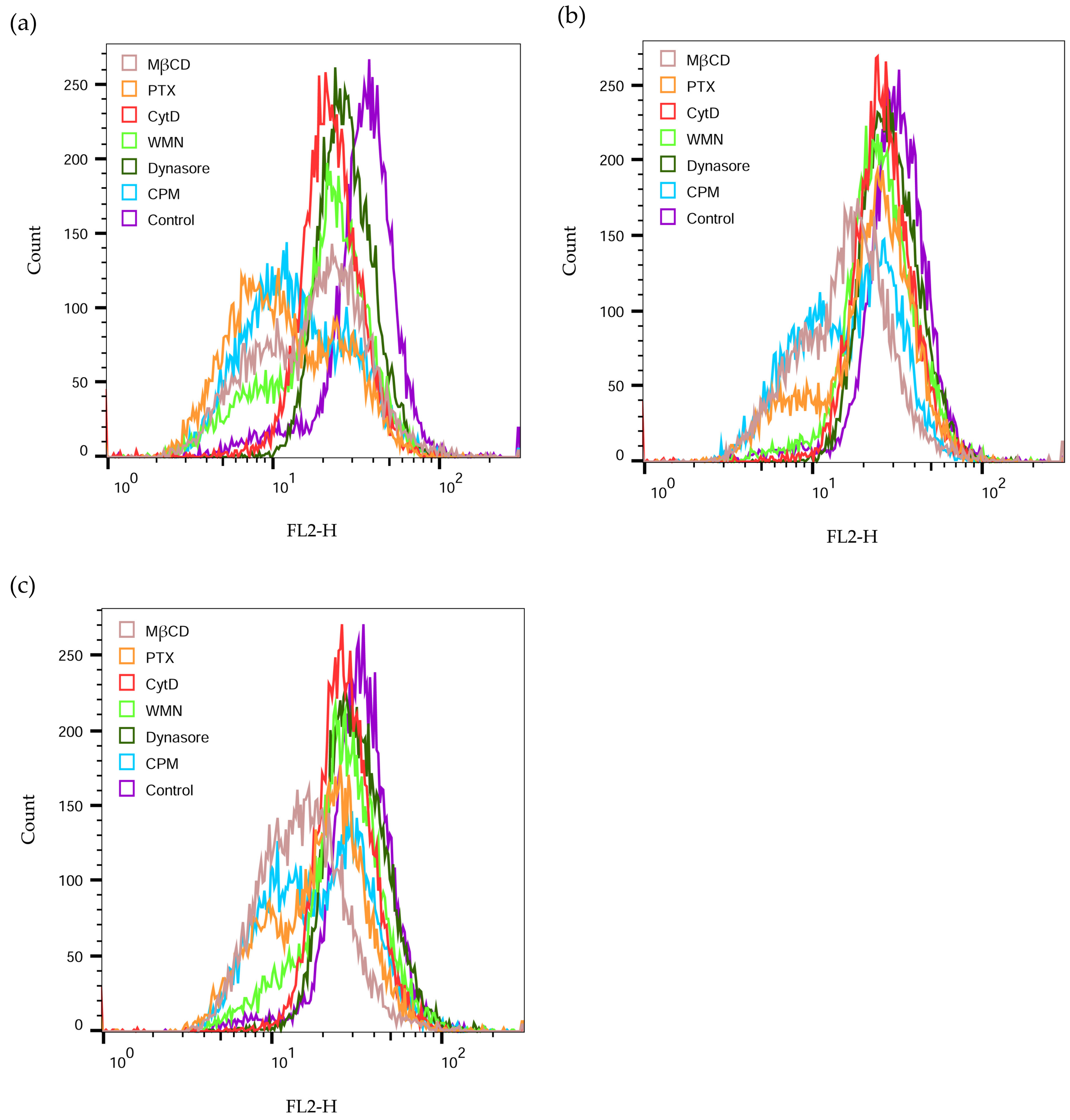

Figure S4a. Flow cytometry analysis for cellular uptake with endocytosis inhibitors of (a) F1, (b) F3, (c) F4 in MCF-7.

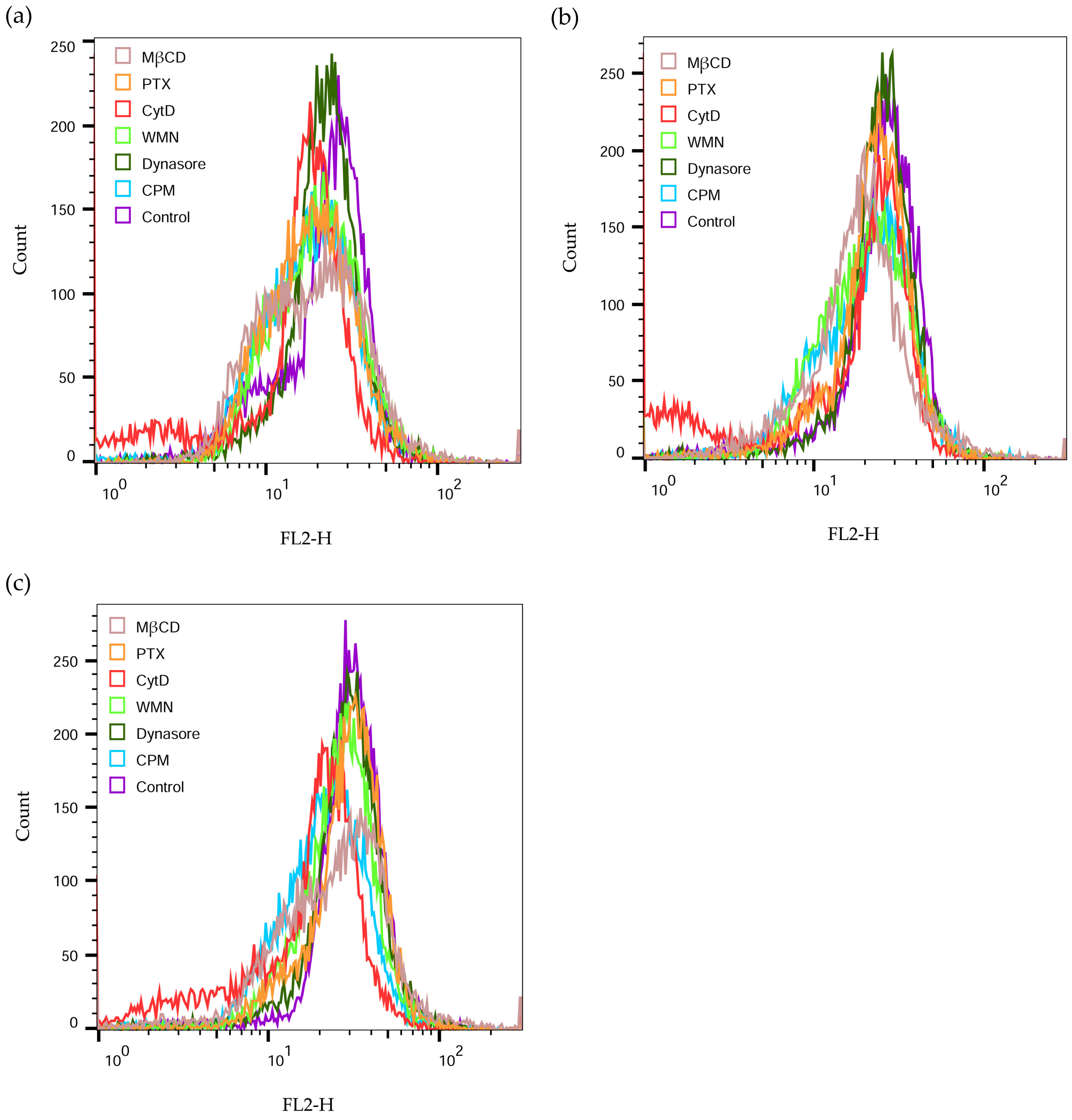

Figure S4b. Flow cytometry analysis for cellular uptake with endocytosis inhibitors of (a) F1, (b) F3, (c) F4 in MDA-MB-231.

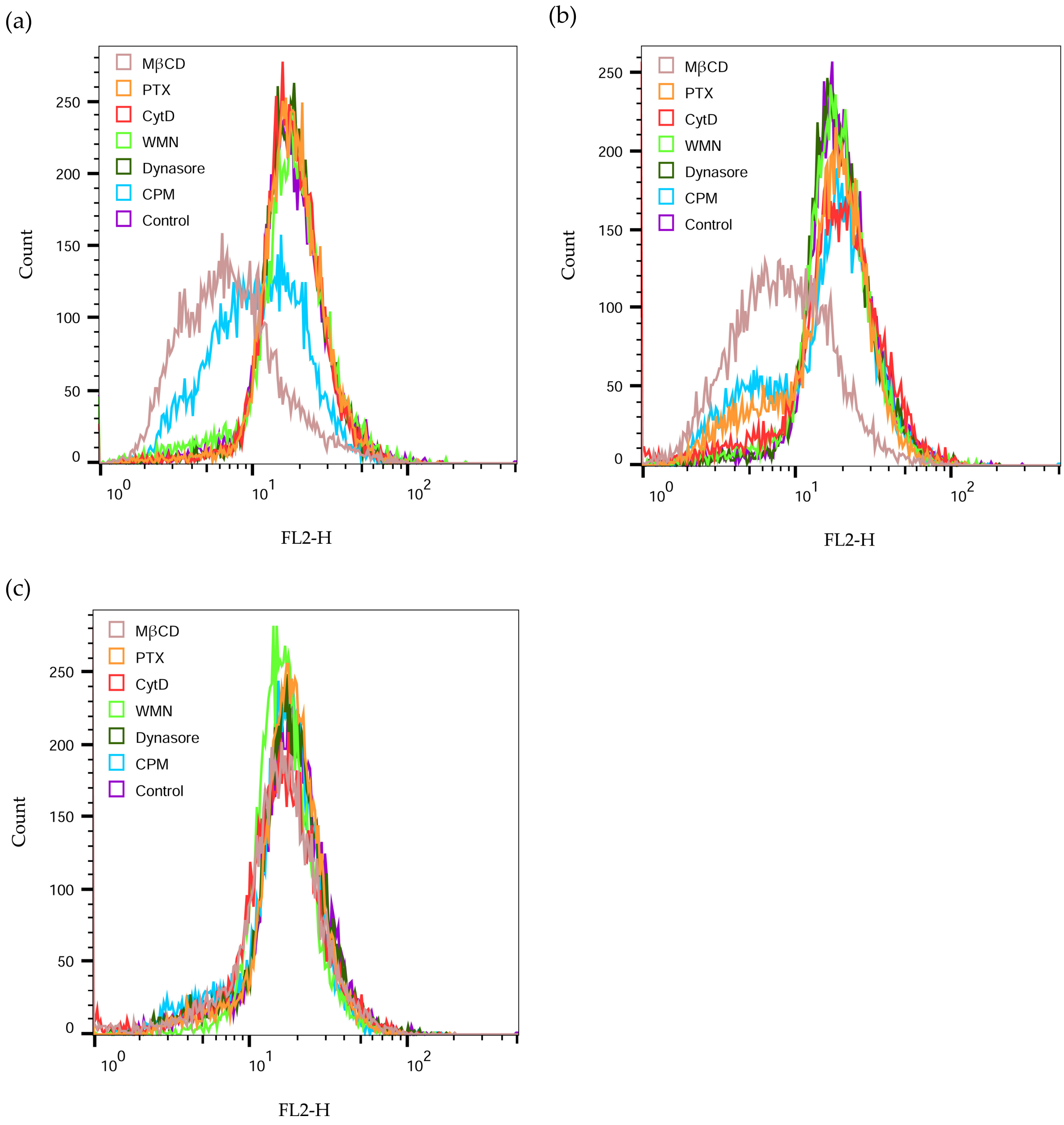

Figure S4c. Flow cytometry analysis for cellular uptake with endocytosis inhibitors of (a) F1, (b) F3, (c) F4 in MDA-MB-468.
